# Sinks for Inorganic Nitrogen Deposition in Forest Ecosystems with Low and High Nitrogen Deposition in China

**DOI:** 10.1371/journal.pone.0089322

**Published:** 2014-02-20

**Authors:** Wenping Sheng, Guirui Yu, Huajun Fang, Chunming Jiang, Junhua Yan, Mei Zhou

**Affiliations:** 1 Key Laboratory of Ecosystem Network Observation and Modeling, Institute of Geographic Sciences and Natural Resources Research, Chinese Academy of Sciences, Beijing, PR China; 2 Institute of Applied Ecology, Chinese Academy of Sciences, Shenyang, PR China; 3 South China Botany Garden, Chinese Academy of Science, Guangzhou, PR China; 4 College of Ecology and Environmental Science, Inner Mongolia Agricultural University, Hohhot Inner Mongolia, PR China; Institute of Botany, Chinese Academy of Sciences, China

## Abstract

We added the stable isotope ^15^N in the form of (^15^NH4)_2_SO_4_ and K^15^NO_3_ to forest ecosystems in eastern China under two different N deposition levels to study the fate of the different forms of deposited N. Prior to the addition of the ^15^N tracers, the natural ^15^N abundance ranging from −3.4‰ to +10.9‰ in the forest under heavy N deposition at Dinghushan (DHS), and from −3.92‰ to +7.25‰ in the forest under light N deposition at Daxinganling (DXAL). Four months after the tracer application, the total ^15^N recovery from the major ecosystem compartments ranged from 55.3% to 90.5%. The total ^15^N recoveries were similar under the (^15^NH4)_2_SO_4_ tracer treatment in both two forest ecosystems, whereas the total ^15^N recovery was significantly lower in the subtropical forest ecosystem at DHS than in the boreal forest ecosystem at DXAL under the K^15^NO_3_ tracer treatment. The ^15^N assimilated into the tree biomass represented only 8.8% to 33.7% of the ^15^N added to the forest ecosystems. In both of the tracer application treatments, more ^15^N was recovered from the tree biomass in the subtropical forest ecosystem at DHS than the boreal forest ecosystem at DXAL. The amount of ^15^N assimilated into tree biomass was greater under the K^15^NO_3_ tracer treatment than that of the (^15^NH4)_2_SO_4_ treatment in both forest ecosystems. This study suggests that, although less N was immobilized in the forest ecosystems under more intensive N deposition conditions, forest ecosystems in China strongly retain N deposition, even in areas under heavy N deposition intensity or in ecosystems undergoing spring freezing and thawing melts. Compared to ammonium deposition, deposited nitrate is released from the forest ecosystem more easily. However, nitrate deposition could be retained mostly in the plant N pool, which might lead to more C sequestration in these ecosystems.

## Introduction

During the last few decades, atmospheric nitrogen (N) deposition has increased sharply as a result of the consumption of fossil fuels, the emission of industrial waste gases, the excessive application of fertilizers, and the rapid development of animal husbandry [1]. The anthropogenic reactive N emission has increased from 15 Tg N a^−1^ in 1860 to 165 Tg N a^−1^ in early 1990 s, and more that 70% of the reactive N deposited back to the terrestrial and aquatic ecosystems [2]. The current global atmospheric deposition of N is approximately 25 to 40 Tg N a^−1^ [3] and is expected to double in the next 25 years [4]. N deposition, as well as increasing concentration of carbon dioxide and ongoing land use/land cover change, has been the well documented issues of global change, altering the biogeochemistry of ecosystems [5].

There has been widespread concern about the effect of increasing N deposition on natural forest ecosystems [6], because of the high sensitivity of biodiversity and productivity of these ecosystems to N input [7]. As one of the most important N sources in forest ecosystem, N deposition increasing might lead to remarkable effects on the forest ecosystem N cycling. Chronic atmospheric N deposition can alter N transmission and transformation processes in the forest ecosystem, such as plant absorption [8], microbe immobilization [9], mineralization [10], nitrification [11], as well as volatilization and leaching losses [12], [13].

The increase in available N resulting from atmospheric N deposition could also stimulate carbon (C) sequestration in terrestrial ecosystems and may provide a reasonable explanation for the CO_2_ “missing sink” [14], [15]. The role of N deposition in determining how strongly a forest acts as a sink for CO_2_ depends on where the deposited nitrogen is immobilized in the ecosystem [16]. The C:N mass ratio in woody tissues is markedly higher than in soil layers, and the turnover time of woody tissues is also longer than that of soils. Therefore, the more deposited N is immobilized in woody tissues, the more C sequestration occurs. In contrast, when more deposited N is immobilized in soil layers, more nitrate leaching and escape of N gases will occur [17], [18].

In several studies, the N deposition levels of forest ecosystems were manipulated to evaluate the complex interactions of processes in the N cycle and to measure N cycling within these ecosystems [19], [20]. However, in these studies, it is difficult to identify how retention of N deposition is distributed among forest ecosystem components. The N stable isotope tracing method provides a good indicator of the fate and retention of N inputs in forest ecosystems [21], [22]. Labeling an N input flux with the stable isotope ^15^N makes it possible to follow the pathways of N that moves through the ecosystem to assess the fate of N deposition across time scales [23]. Numerous ^15^N tracing experiments have been conducted in forest ecosystems in Europe and the United States [18].

In China, reactive N emissions have increased more than threefold in the past 30 years, and the N deposition rate shows a higher mean value than is found in the United States and Europe, because of the astonishing economic growth that has occurred since the early 1980 s [24], [25]. And, there are large spatial differences in the intensity of N deposition among China, owing to unbalanced development in different regions. In eastern China, the amount of N deposited in forest ecosystems in the form of dissolved inorganic nitrogen ranges from 1.3 to 29.5 kg N ha^−1^ a^−1^, and the nitrate to ammonium ratio also changes under different intensities of N deposition [26]. In the forest ecosystems suffering heavy N deposition in southern China, dissolved inorganic and organic N have been detected in the surface runoff and soil solution [19].

In this study, our objective was to examine the different fate of N deposition in two old forest ecosystems, by tracing the nitrate and ammonium forms of deposited N by (^15^NH_4_)_2_SO_4_ and K^15^NO_3_ tracers. We hypothesized that less recovery of ^15^N and lower N assimilation by trees would occur in the N abundant subtropical forest ecosystem than in the N limited boreal forest ecosystem due to the more open N cycle there. We also hypothesized that the recovery of ^15^N would be higher with the ammonium treatment due to the greater mobility of nitrate.

## Materials and Methods

### Site Description

We chose two typical old-growths forests to characterize the differences in N deposition residing in these systems and the different contribution of N deposition to C sequestration under different N deposition intensities in eastern China (Table 1). N deposition in the subtropical broad-leaved forest at Dinghushan (DHS) was observed to be as high as 29 kg N ha^−1^ a^−1^, and N saturation occurred in this forest ecosystem [19]. In contrast, the boreal coniferous forest at Daxinganling (DXAL) shows low available N, with nitrogen deposition of only 1.8 kg N ha^−1^ a^−1^. The predominant species found in the tree layers in the subtropical monsoon evergreen broad-leaved forest are *Schima superba*, *Syzygium jambos*, and *Castanopsis chinensis* [27]. The boreal coniferous forest at DXAL is pure forest in which *Larix gmelinii* is the predominant tree species [28]. The soils in the two old-growth forests in the south and the north are Greyzems and Ferralsols, respectively. A more extensive description of these sites is given in Table 1.

**Table 1:** Stand characteristics and surface soil (0–20 cm) properties at the two old-growth forest sites.

Sites	Dinghushan(DHS)	Daxinganling (DXAL)
Forest type	Subtropical evergreen broadleaved forest	Boreal coniferous forest
State age (yr)	400	180
Location	23°10′N, 112°34′E	50°56′N, 121°30′E
Elevation (m)	300	810
Mean annual temperature (°C)	20.9	−5.4
Mean annual precipitation (mm)	1 564	500
N deposition (kgN ha^−1^ yr^−1^)	29.5	1.8
Net N mineralization (kg N ha^−1^ yr^−1^)	164.1	71.7
Biomass (Mg C ha^−1^)	87.7(8.7)	56.1(4.8)
Litter input (Mg C ha^−1^ yr^−1^)	8.42(0.47)	2.50(0.27)
Gravel (0.2–2 mm, %)	34.30	11.16
Silt (0.02–0.2 mm, %)	19.65	51.76
Sand (0.002–0.02 mm, %)	19.65	27.55
Clay (<0.002 mm, %)	26.22	9.53
SOC density(kg m^−2^)	8.8(0.58)	14.62(0.35)
Soil pH	3.80(0.11)	6.03(0.09)

Data source: Chinese Ecosystem Research Network (CERN) database.

### Experimental Design

In May 2008, six separate plots (9 × 9 m) were randomly distributed at each forest research station, aside the long-term forest ecosystem observation plot, with buffer zones of at least five meters. In three of the plots, a ^15^N tracer in the form of 99% enriched (^15^NH4)_2_SO_4_ was added. In the other plots, the applied ^15^N tracer was in the form of 99% enriched K^15^NO_3_. The quantity of the ^15^N tracer applied to each plot was calculated to be 36 mg ^15^N m^−2^, thus increasing the ambient ^15^N concentrations in these ecosystem pools significantly above natural abundance levels while minimizing the disturbance of the soil inorganic N pools. The ^15^N tracers were dissolved in deionized water and added to each plot in a single application using a portable sprayer. The control plots for the labeling experiment were situated within the long-term fixed observation plots. Plant and soil samples were collected before and 1 wk, 1 mo, and 4 mo after the addition of^ 15^N. Considering that the intensity of N mobilization is strongest during the spring melt, we collected an additional sample one year after the application of ^15^N in the boreal forest ecosystem at DXAL.

### Sampling and Analysis

By the permissions of Dinghushan Nature Reserve Administration and Daxinganling Hanma Nature Reserve Administration, tree foliage, bark, and wood samples were collected from three each kind of dominant trees within each plot in the two forest ecosystems. Living fine roots (intact and fibrous) were separated from Oa+e horizon samples and the 0–10 and 10–20 cm mineral soil layers. Organic soil samples, consisting of the upper Oi horizon and lower Oe+a horizon, were collected from three random (20×20 cm) areas within each plot. Three sets of mineral soil profile samples were collected from three depths (0–10, 10–20, and 20–40 cm) with a 5 cm-diameter auger.

Plant and litter samples were dried to a constant weight at 65°C. Mineral soils were air dried at room temperature and then sieved through a 2 mm sieve. All samples were ground into a fine powder with a planetary mill and passed through a no. 100 mesh sieve, then stored in glassware. Plant and soil samples were oven dried at 65°C for 24 h and cooled in an evacuated desiccator immediately prior to analysis.

C and N concentrations and *δ* ^15^N values were determined simultaneously with an automatic online elemental analyzer (Flash EA1112, ThermoFinnigan, Milan, Italy) coupled to an isotope ratio mass spectrometer (IRMS) (Finnigan MAT 253, Thermo Electron, Bremen, Germany). The standard deviation of 10 repeated samples was <0.4‰.

### Calculations and Statistics

We estimated the movements of the ^15^N tracers within forest components using an ecosystem N pool size based on field measurements, changes in the ^15^N contents of the ecosystem pools following tracer addition, and ^15^N mass recoveries.

The percentage of the total amount of tracer applied to the plots recovered within the pools was calculated as follows:
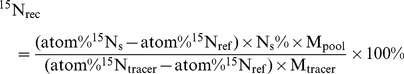
(1)where ^15^N_rec_ is the percent recovery of the ^15^N tracer recovered in the labeled N pool (%); atom%^15^N_s_ is the atom% of ^15^N in the labeled N sample; atom%^15^N_ref_ is the atom% of ^15^N in the reference (pre- or non-labeled) N pool; atom%^15^N_trance_ is the atom% of ^15^N in the applied tracer; N_s_% is the N concentration of the labeled N sample; M_pool_ is the dry mass of the labeled pool (g m^−2^); and M_tracer_ is the mass of ^15^N in the ^15^N tracer applied to a plot (g m^−2^).

The differences in the *δ* ^15^N and N concentrations in the samples were tested through analysis of variance (ANOVA). All analyses were conducted using the SPSS software package (SPSS for Windows, Version 13.0, Chicago, IL, USA). The level of statistical significance was set at *p*<0.05 unless stated otherwise.

## Results

### Natural ^15^N Abundances and Ecosystem N Pools

Prior to the addition of the ^15^N tracers, the natural ^15^N abundance in the ecosystem N pools ranged from −3.4‰ to +10.9‰ in the subtropical forest ecosystem and from −3.92‰ to +7.3‰ in the boreal forest ecosystem. The natural ^15^N abundance exhibited the same increasing tendency in different ecosystem N pools. The *δ* ^15^N values increased as follows in the tree N pools: wood>roots>foliage>branches and increased from the forest floor to the deep mineral soils (Table 2). While the mean S.E. did not exceed 0.8‰ in nearly all of the N pools, the natural ^15^N abundances in the two forest ecosystems were well defined with respect to their isotopic compositions.

**Table 2:** Natural ^15^N abundance and N mass in the major ecosystem pools in the two old growth forest ecosystems.

	Subtropical forest ecosystem (DHS)				Boreal forest ecosystem (DXAL)			
Ecosystem pool	Natural ^15^N abundance	Mass^a^	N	C/N	Natural ^15^N abundance	Mass^a^	N	C/N
	(‰)	(kg/ha)	(kg/ha)		(‰)	(kg/ha)	(kg/ha)	
Tree								
Foliage	−2.26(0.25)	8,483	163.9	23.8	−3.92(0.34)	2,039	69.2	15.2
Branches	−3.08(0.25)	86,699	934.7	40.3	−4.99(0.21)	11,874	50.4	122.4
Wood	−1.22(0.38)	196,740	469.6	183.5	−1.20(0.56)	86,197	66.9	705.1
Roots	0.82(0.44)	59,312	787.3	33.7	9.88(1.46)	22,266	112.9	108.8
Forest floor								
Oi	−2.49(0.26)	7,612	145.4	26.3	−0.93(0.04)	5,526	71.8	35.0
Oa+e	−2.90(0.15)	12,796	147.2	33.7	−1.26(0.18)	5,557	74.1	35.9
Mineral soil								
0–10 cm	6.21(0.49)	861,000	2 000.0	18.8	4.34(0.27)	151,333	1 057.7	30.1
10–20 cm	8.91(0.20)	974,000	1 109.4	23.3	6.41(0.22)	748,400	1 024.9	21.0
Total	–	–	5 757.4	–	–	–	2 527.8	–

a: From a database of the Chinese Ecosystem Research Network (CERN).

The ecosystem N pool of the subtropical forest ecosystem consisted of 5.76 Mg N (excluding shrubs, herbage, and soils >20 cm deep), which was more than twice the size of the N pool found in the boreal coniferous forest (Table 2). The mineral soils contained the greatest amounts of N in both forest ecosystems. The N pool of the 0–20 cm soil layer accounted for 54% of the total N in the subtropical forest ecosystem, and this proportion was more than 80% in the boreal forest ecosystem. In the tree pools, the total N pools were relatively large, accounting for more than 40% of the total N pool in the subtropical forest ecosystem, whereas this proportion was only 11.8% in the boreal forest ecosystem. The forest floor pool accounted for only 5.1% and 5.8% of the total of measured N pools in the subtropical and the boreal forests, respectively.

### Total ^15^N Recovery

**Figure 1:**
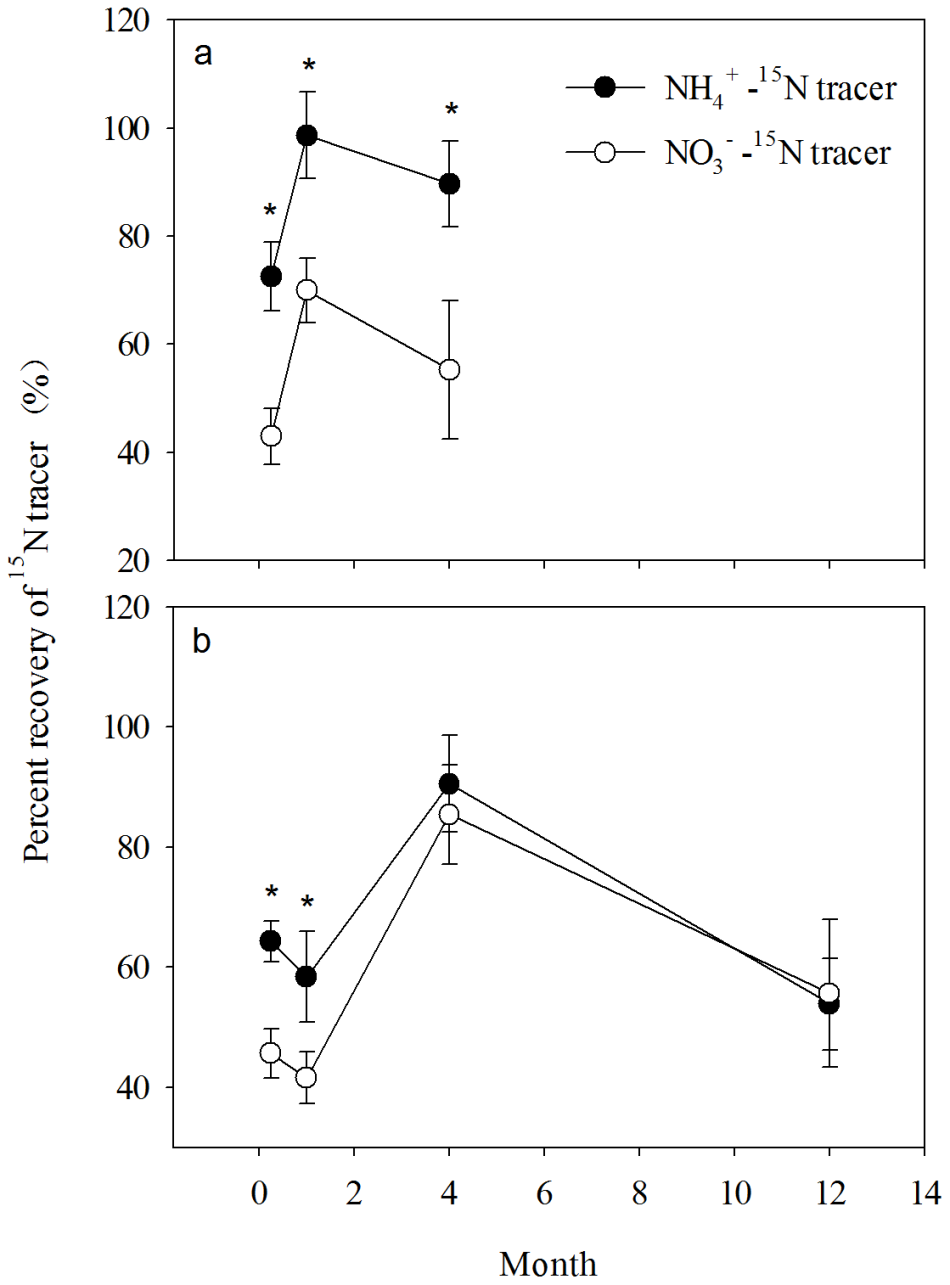
Changes in total ^15^N recovery rates in the subtropical forest ecosystem at DHS (a) and in the boreal forest ecosystem at DXAL (b) in the (^15^NH4)_2_SO_4_ and K^15^NO_3_ tracer treatments at one week, one month, four months, and one year (sampled only at DXAL) after the addition of the ^15^N tracers. An asterisk(*)means that there is a significant difference in ^15^N recovery rates between the two tracer treatments (*p*<0.05).

In the subtropical forest ecosystem, the average total ^15^N recovery was 71.9% one week after the addition of the (^15^NH_4_)_2_SO_4_ tracer, and the recovery rapidly rose within one month and decreased after the rapid growth season. The same fluctuation of the ^15^N recovery was observed following K^15^NO_3_ treatment (Fig. 1a). The total ^15^N recoveries were much higher following treatment with the (^15^NH_4_)_2_SO_4_ tracer than the K^15^NO_3_ tracer, and significant differences were detected for all of the three sampling times (Fig. 1a).

In the boreal forest ecosystem, the total recovery of the applied ^15^N declined a little in the first month, but rose to 90.5% after four months. The total recovery rate decreased back again one year later (Fig. 1b). As in the subtropical forest ecosystem, the fluctuation of ^15^N recovery was same in the K^15^NO_3_ tracer treatment, and there were also significant differences in total ^15^N recovery rates between the two tracers’ treatments. However, these differences disappeared four months later in the boreal forest ecosystem (Fig. 1b).

### N Deposition Retention

Based on the ^15^N recovery in each N pool and the N deposition flux measured in the forest ecosystem, the N deposition retention was calculated. More than three quarters of deposited N was retained in the forest ecosystem after the growing season or the rapid growth season, because ammonium immobilization rate was high and a large proportion of N deposition was in the form of ammonium in China (Table 3). Even after the spring freezing and thawing melts, there was still more than half part of deposited N was retained in the forest ecosystem.

**Table 3:** Nitrogen deposition and retention in the two old growth forest ecosystems during the experimental period.

	Nitrogen deposition^a^		Immobilization rate^b^			Nitrogen deposition
	kg N/(ha·a)		(%)			retention
	Ammonium	Nitrate	Ammonium		Nitrate	%
Subtropical evergreen forest (DHS)	6.9	4.9	89.7	55.3		75**.**4
Boreal coniferous forest (DXAL)	0.7	0.3	90.5	85.4		88.9

a Inorganic nitrogen deposition from May to August 2008, from a database of the Chinese Ecosystem Research Network (CERN).

b Total ^15^N recovery in the ecosystem four months after the application of the (^15^NH_4_)_2_SO_4_ and K^15^NO_3_ tracers.

### ^15^N Recovery in each N Pool

**Figure 2:**
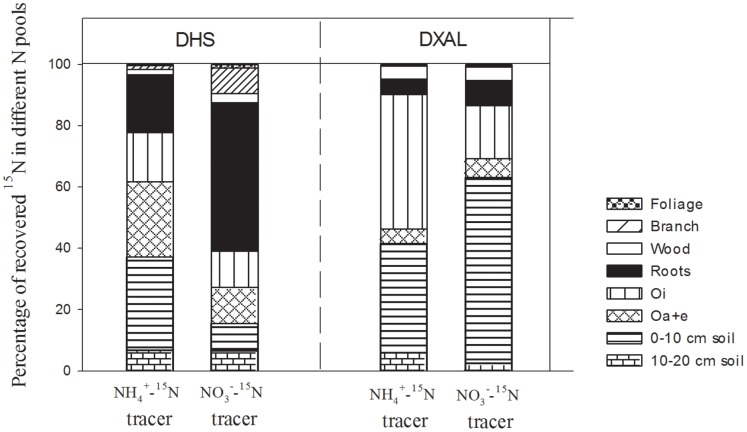
Percentage of recovered ^15^N in different ecosystem N pools in the subtropical forest ecosystem at DHS and in the boreal forest ecosystem at DXAL in the (^15^NH4)_2_SO_4_ and K^15^NO_3_ application treatments.

The fate of the added ^15^N was significantly different between the two tracers’ treatments in the subtropical forest ecosystem. Four months after (^15^NH_4_)_2_SO_4_ tracer addition, the organic soil layer (comprised of the upper Oi horizon and lower Oe+a horizon) was found to be the largest sink. The second is the mineral soil layer, and tree N pool accounted for less than one third of the total recovered^ 15^N tracer (Fig. 2). In contrast, following treatment with the K^15^NO_3_ tracer, the largest sink was the tree N pool, and the recovered^ 15^N tracer in both organic soil layer and mineral soil layer was less than 40% (Fig. 2).

In the boreal forest, the trees contained the minimum ^15^N pool four months after ^15^N tracer application in both the (^15^NH_4_)_2_SO_4_ and K^15^NO_3_ tracer treatments. The difference between the treatments using the two different tracers was because that the organic soil layer represented the largest ^15^N sink in the (^15^NH_4_)_2_SO_4_ tracer treatment, while the mineral soil layer was the largest ^15^N sink in the K^15^NO_3_ tracer treatment (Fig. 2).

### Impact of Spring Melt on ^15^N Retention

**Figure 3:**
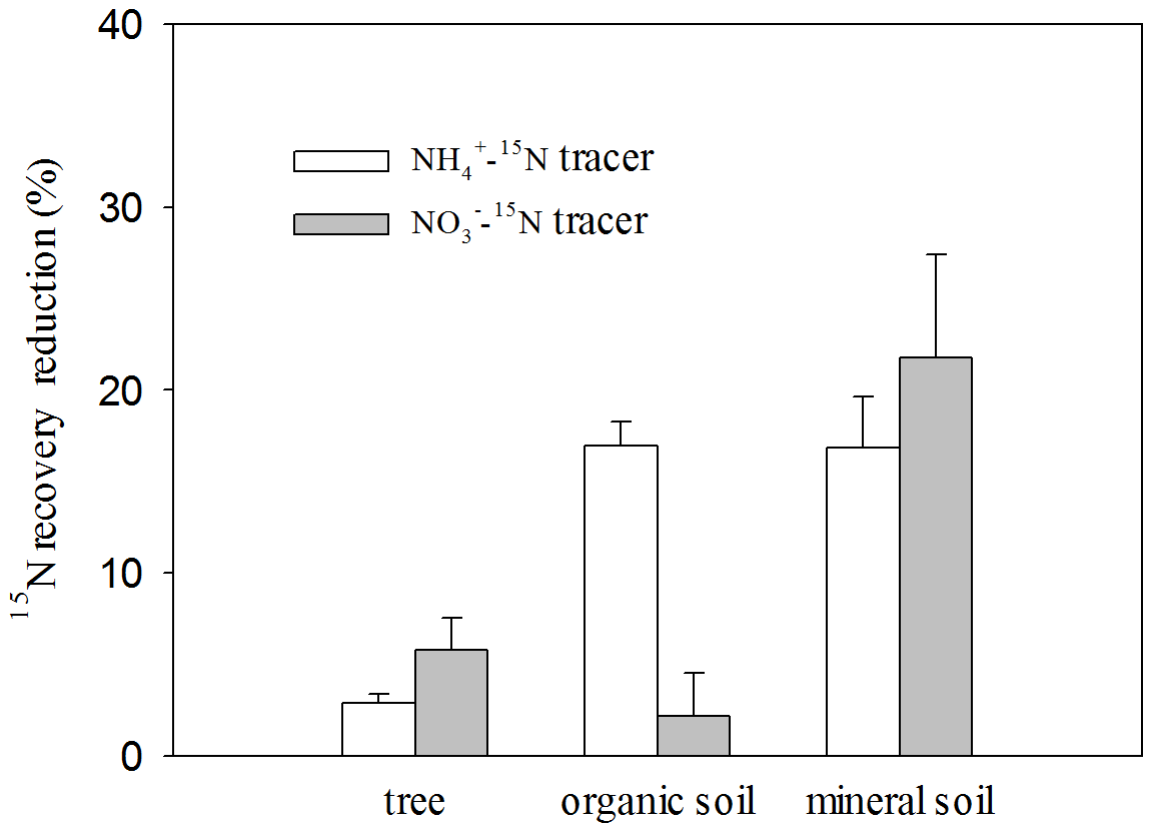
^15^N recovery reduction after the spring freezing and thawing melts in the boreal forest ecosystem at DXAL in the (^15^NH4)_2_SO_4_ and K^15^NO_3_ application treatments. An asterisk(*)means that there is a significant difference in the change of ^15^N recovery rates between the two tracer treatments (*p*<0.05).

After the repeated freezing and thawing cycles in spring, total ^15^N recovery decreased by 36.7% and 29.8% under the (^15^NH_4_)_2_SO_4_ and K^15^NO_3_ tracer treatments, respectively, in the boreal forest ecosystem (Fig. 1). The reduction of retained ^15^N from the forest ecosystem mainly occurred in the soil layer (Fig. 3). The amount of reduction in the organic soil and the mineral soil were almost same in the (^15^NH_4_)_2_SO_4_ tracer treatment, and there was about 40% of the retained ^15^N losing from each of the two N pools. However, the loss of retained ^15^N mainly occurred in the mineral soil in the K^15^NO_3_ tracer treatment, and the reduction percent is also about 40% in N pool of mineral soil. The absolute amount of ^15^N reduction in the tree N pool is few in both the treatments, but the percentage of the ^15^N loss in the tree N pool was more than 50% in the in the K^15^NO_3_ tracer treatment.

## Discussion

### Natural ^15^N Abundances

In our study, ^15^N natural abundance in the foliage and branches in the subtropical forest was higher than in the boreal forest. Meta-analysis of ^15^N tracer field studies in Europe and North America indicates that foliar ^15^N natural abundance is a good indicator of ecosystem N-retention capacities [18]. This point is also supported by our study in eastern China, for the average total ^15^N tracer recovery rate was significantly higher in the boreal forest ecosystem than in subtropical forest ecosystem, after the growing season or fast growth season (Fig. 1).

However, the ^15^N natural abundance in roots was much greater in the boreal forest than in the subtropical forest (Table 2). The rate of N-cycling, N losses, and mycorrhizal association are the possible reason for the difference of plant ^15^N natural abundance in the terrestrial ecosystems [29]. The inconsistent variation of ^15^N natural abundance indicated that the plants survived the low inorganic N availably mainly by the existence of mycorrhizal fungi in the boreal forest ecosystem. Through the proliferation of their hyphae, mycorrhizal fungi supply their host plants with organic N via protein degradation [30]. The transfer of N from mycorrhizal fungi to their hosts favors ^15^N-depletion because of fractionation during metabolic processes and the selective retention of specific N compounds by the fungi, thus the fungal N is enriched by 3–11‰ relative to the host plant N [29], [31]. Therefore, the ^15^N natural abundance in the foliage and branches was relatively low, whereas the ^15^N natural abundance in infected roots was considerably higher in the boreal forest ecosystem.

### N Deposition Retention

In our study, the average total ^15^N tracer recovery rate was significantly higher in the boreal forest ecosystem than in subtropical forest ecosystem (Fig. 1, Table. 3). The ^15^N recovery rate was significantly higher in the (^15^NH_4_)_2_SO_4_ treatment than in the K^15^NO_3_ treatment, especially at the beginning of the experiment (Fig. 1). At the time N deposition entering the forest ecosystem, ammonium is likely retained on cation exchange sites in soil organic matter and clays, while nitrate is more mobile and prone to uptake by plants and leaching or gaseous losses during denitrification [32], [33]. Therefore, more ^15^N tracer was recovered in the (^15^NH_4_)_2_SO_4_ treatment, but was mainly stored in the organic and mineral soil layers. However, the less ^15^N tracer was recovered in the K^15^NO_3_ treatment, but more was utilized by trees (Fig. 2). Because the ammonium recovery rate is higher and the N deposition was mainly in ammonium in the forest ecosystem in China [26], more than three quarter of the N deposition could be retained in the forest ecosystem in the growing or fast growth season (Table 3).

### Reasons for N Retention Difference in the Two Forest Ecosystem

As we hypothesized, the ^15^N recovery rate was continuously lower in the tropical forest ecosystem than in the boreal forest ecosystem after the growing season or the fast growth season (Fig. 1). The deposited N partly was absorbed by plants and by microbes, partly was immobilized in the soil through abiotic way, and partly escaped from the ecosystem by leaching and gas emission. Higher temperature in the tropical forest ecosystem might lead to greater rates of N uptake by plants and microbes, while, the rates of soil N-cycling processes, such as mineralization and nitrification, and losses via gas emissions or leaching, were also increased by the higher temperature. And the possible increases in rates of soil N-cycling processes could have more than offset the greater uptake. In the subtropical forest ecosystem, nitrification accounts for close to 80% of the net mineralization, and nitrate is the main N source in this forest ecosystem [34]. Therefore, ^15^N tracer recovery was relatively low in the treatment involving the K^15^NO_3_ tracer application (Fig. 1 Table. 3).

The boreal forest ecosystem was located in northeast China, where microbial activity is restricted by low temperature, and there is far less available N than is needed (Table 1). External N could be tightly fixed in the ecosystem when entering to the ecosystem. The higher soil C:N, which promotes microbial N immobilization and reduces net nitrification, could be another reason contributing to greater^ 15^N recovery rate in the boreal forest. Especially, C:N in the organic soil, strongly influences the nitrate losing in the forest ecosystem [35]. Study across the forest ecosystems of Europe and North America also supports that the total ecosystem retention of applied ^15^N positively correlates with soil C:N ratio [18].

Except for temperature and soil C:N ratio, N deposition is another most consistent and useful indicator of N retention rate [18]. The deposited N from atmosphere accumulated in the ecosystem, and changed the N saturation status of the forest ecosystems [36].The rising of N availability relaxes competition for N, thus stimulating ammonium oxidizers, and leading to elevated production and loss of N from the ecosystem [37]. The boreal forest ecosystem, historically poor in N availability, would have a substantial capacity to store excess N. However, the heavy atmospheric N deposition in the subtropical forest ecosystem reduced biotic demand and increased soil N-cycling processes, and finally led to less ^15^N trace recovered (Fig. 1).Study in the United States also finds that atmospheric N retention is >90% for sites receiving <7 kg N ha^−1^ yr^−1^, which decreases to <60% at sites receiving >11 kg N ha^−1^ yr^−1^ [38].

### Contribution of Deposited N to C Sequestration

Following the growing season or the rapid growth season, a greater amount of N was utilized by tree following K^15^NO_3_ tracer application compared to (^15^NH_4_)_2_SO_4_ tracer application in both forest ecosystems (Fig. 2). This occurred not only because nitrate is more mobile than ammonium but also because nitrate is more important in balancing cation uptake [32], [33]. The utilization of deposited N by the tree in subtropical forest ecosystem was higher than in boreal forest ecosystem (Fig. 2). The difference in N uptake by tree between the treatments involving the two ^15^N tracers was much smaller in the boreal forest ecosystem, likely due to the high ammonia absorption rate under N limited conditions [39], [40].

However, contrary to our hypothesis, the stimulating effects of external N on tree growth were not obvious in the boreal forest ecosystem, most likely due to the difference in tree species and the competition for N between bacteria and vegetation in this N limited ecosystem [41]. Although N saturation has been observed in the subtropical forest ecosystem [19], the utilization of external N by tree was still higher than that in the boreal forest ecosystem. The role of N deposition in determining how strongly a forest acts as a sink for CO_2_ depends on where the deposited nitrogen is immobilized in the ecosystem [18]. The C:N mass ratio in woody tissues is markedly higher than in soil layers, and the turnover time of woody tissues is also longer than that of soils. For the more deposited N is immobilized in woody tissues, the more C sequestration would be stimulated by N deposition in the subtropical forest ecosystem.

### Uncertainties

In this study, we explored where deposited N ultimately resides as well as the role that N deposition plays in contributing to C sequestration in the forest ecosystems of China. However, due to the lack of enough technical and capital supports, there are still some shortcomings that need to be overcome in the future. First of all, the ^15^N tracers were sprayed on the forest floor in this study, giving no considerations to the possibility of deposited N absorption by foliage. Therefore, the utilization of deposited N might have been underestimated in this study, because previous studies show that nitrogen can be absorbed by tree canopies from atmospheric input, and this process relates to both ammonium and nitrate ions [42]–[44]. Secondly, only foliage, bark, and wood of dominant trees, as well as organic layer and mineral soil above 20 cm were sampled within each plot. Given the possible immobilization of deposited N by other plants and in the deep soil layers, the utilization of deposited N by the forest ecosystems might have been underestimated to some extent in this study. Finally, less sampling frequency and shorter duration of the experiment might also be the source of uncertainty in this study. These all need to be perfected in the future studies.

### Conclusions

This study traced atmospheric ammonium and nitrate deposition under different N deposition levels via the application of (^15^NH4)_2_SO_4_ and K^15^NO_3_ in two forest ecosystems in China. Prior to ^15^N tracer application, the natural ^15^N abundance showed the same pattern in different ecosystem N pools in these two forest ecosystems, with the following order found in the tree N pools: wood>roots>foliage>branches and with increases being observed from the forest floor to deep mineral soils. Four months after the addition of the^ 15^N tracers, the ^15^N recovery rates in the subtropical forest ecosystem at DHS were 89.7% and 55.3% under (^15^NH4)_2_SO_4_ and K^15^NO_3_ application, respectively. The ^15^N recovery rates were both higher in the two tracer treatments, with recovery rates of 90.5% and 85.4% being observed, respectively. The amounts ^15^N recovered in the tree pools were significantly greater in the subtropical forest ecosystem, accounting for 22.3% and 61.0% of the total recovered^ 15^N pools under the (^15^NH4)_2_SO_4_ and K^15^NO_3_ tracer treatments, respectively. However, in the boreal coniferous forest at DXAL, the trees contained the smallest ^15^N pool four months after ^15^N application, accounting for 9.8% and 13.4% of the total recovered^ 15^N pools under the (^15^NH4)_2_SO_4_ and K^15^NO_3_ tracer treatments, respectively. Our findings suggest that more than half of atmospheric N deposition is immobilized in the forest ecosystems of China, even in areas under a heavy N deposition intensity and in ecosystems undergoing spring freezing and thawing melts. Although deposited nitrate is readily released from forest ecosystems, nitrate deposition is immobilized more in tree biomass, which means that it would do more contributions to C sequestration than ammonia deposition in the forest ecosystems of China.

## References

[1] Galloway JN, Townsend AR, Erisman JW, Bekunda M, Cai ZC, et al (2008) Transformation of the nitrogen cycle: Recent trends, questions, and potential solutions. Science 320: 889–892. 18487183 10.1126/science.1136674

[2] Galloway JN, Dentener FJ, Capone DG, Boyer EW, Howarth RW, et al (2004) Nitrogen cycles: past, present, and future. Biogeochemistry 70: 153–226.

[3] Neff JC, Chapin III FS, Vitousek PM (2003) Breaks in the cycle: dissolved organic nitrogen in terrestrial ecosystems. Frontiers in Ecology and the Environment 1: 205–211.

[4] Lamarque JF, Kiehl J, Brasseur G, Butler T, Cameron-Smith P, et al.. (2005) Assessing future nitrogen deposition and carbon cycle feedback using a multimodel approach: Analysis of nitrogen deposition. Journal of Geophysical Research: Atmospheres (1984–2012) 110.

[5] Vitousek PM (1994) Beyond global warming: ecology and global change. Ecology 75: 1861–1876.

[6] Klopatek JM, Barry MJ, Johnson DW (2006) Potential canopy interception of nitrogen in the Pacific Northwest, USA. Forest ecology and management 234: 344–354.

[7] Aber JD, Magill AH (2004) Chronic nitrogen additions at the Harvard Forest (USA): the first 15 years of a nitrogen saturation experiment. Forest Ecology and Management 196: 1–5.

[8] Magill AH, Aber JD, Berntson GM, McDowell WH, Nadelhoffer KJ, et al (2000) Long-term nitrogen additions and nitrogen saturation in two temperate forests. Ecosystems 3: 238–253.

[9] Tietema A (1998) Microbial carbon and nitrogen dynamics in coniferous forest floor material collected along a European nitrogen deposition gradient. Forest Ecology and Management 101: 29–36.

[10] Chen Y, Högberg P (2006) Gross nitrogen mineralization rates still high 14 years after suspension of N input to a N-saturated forest. Soil Biology and Biochemistry 38: 2001–2003.

[11] Venterea RT, Groffman PM, Verchot LV, Magill AH, Aber JD (2004) Gross nitrogen process rates in temperate forest soils exhibiting symptoms of nitrogen saturation. Forest ecology and management 196: 129–142.

[12] Fang Y, Gundersen P, Mo J, Zhu W (2009) Nitrogen leaching in response to increased nitrogen inputs in subtropical monsoon forests in southern China. Forest Ecology and Management 257: 332–342.

[13] Hoegberg P, Fan H, Quist M, Binkley D, Tamm CO (2006) Tree growth and soil acidification in response to 30 years of experimental nitrogen loading on boreal forest. Global Change Biology 12: 489–499.

[14] Gifford R (1994) The global carbon cycle: a viewpoint on the missing sink. Functional Plant Biology 21: 1–15.

[15] Luo Y, Hui D, Zhang D (2006) Elevated CO2 stimulates net accumulations of carbon and nitrogen in land ecosystems: a meta-analysis. Ecology 87: 53–63. 16634296 10.1890/04-1724

[16] Magnani F, Mencuccini M, Borghetti M, Berbigier P, Berninger F, et al (2007) The human footprint in the carbon cycle of temperate and boreal forests. Nature 447: 849–851. 10.1038/nature05847 17568744

[17] Nadelhoffer KJ, Emmett BA, Gundersen P, Kjønaas OJ, Koopmans CJ, et al (1999) Nitrogen deposition makes a minor contribution to carbon sequestration in temperate forests. Nature 398: 145–148.

[18] Templer P, Mack M, III FC, Christenson L, Compton J, et al (2012) Sinks for nitrogen inputs in terrestrial ecosystems: a meta-analysis of 15N tracer field studies. Ecology 93: 1816–1829. 22928411 10.1890/11-1146.1

[19] Fang Y, Gundersen P, Mo J, Zhu W (2008) Input and output of dissolved organic and inorganic nitrogen in subtropical forests of South China under high air pollution. Biogeosciences 5: 339–352.

[20] Wright RF, Rasmussen L (1998) Introduction to the NITREX and EXMAN projects. Forest Ecology and Management 101: 1–7.

[21] Schlesinger WH (2009) On the fate of anthropogenic nitrogen. Proceedings of the National Academy of Sciences 106: 203–208. 10.1073/pnas.0810193105 PMC2613040 19118195

[22] Turner MM, Henry HA (2009) Interactive effects of warming and increased nitrogen deposition on 15N tracer retention in a temperate old field: seasonal trends. Global Change Biology 15: 2885–2893.

[23] Currie WS, Nadelhoffer KJ (1999) Original Articles: Dynamic Redistribution of Isotopically Labeled Cohorts of Nitrogen Inputs in Two Temperate Forests. Ecosystems 2: 4–18.

[24] Liu X, Zhang Y, Han W, Tang A, Shen J, et al (2013) Enhanced nitrogen deposition over China. Nature 494: 459–462. 23426264 10.1038/nature11917

[25] Lv C, Tian H (2007) Spatial and temporal patterns of nitrogen deposition in China: Synthesis of observational data. Journal of Geophysical Research: Atmospheres (1984–2012) 112.

[26] Sheng W, Yu G, Jiang C, Yan J, Liu Y, et al (2013) Monitoring nitrogen deposition in typical forest ecosystems along a large transect in China. Environmental monitoring and assessment 185: 833–844. 22411032 10.1007/s10661-012-2594-0

[27] Mo J, Zhang W, Zhu W, Gundersen P, Fang Y, et al (2008) Nitrogen addition reduces soil respiration in a mature tropical forest in southern China. Global Change Biology 14: 403–412.

[28] Fan P, Jiang Y (2010) Nitrogen dynamics differed among the first six root branch orders of Fraxinus mandshurica and Larix gmelinii during short-term decomposition. Journal of plant research 123: 433–438. 20082111 10.1007/s10265-009-0303-z

[29] Högberg P (1997) Tansley review No 95 - N-15 natural abundance in soil-plant systems. New Phytologist 137: 179–203. 10.1046/j.1469-8137.1997.00808.x 33863175

[30] Hobbie EA, Hobbie JE (2008) Natural abundance of (15)N in nitrogen-limited forests and tundra can estimate nitrogen cycling through mycorrhizal fungi: A review. Ecosystems 11: 815–830.

[31] Högberg P, Högbom L, Schinkel H, Högberg M, Johannisson C, et al (1996) N-15 abundance of surface soils, roots and mycorrhizas in profiles of European forest soils. Oecologia 108: 207–214. 28307831 10.1007/BF00334643

[32] Koba K, Hirobe M, Koyama L, Kohzu A, Tokuchi N, et al (2003) Natural N-15 abundance of plants and soil N in a temperate coniferous forest. Ecosystems 6: 457–469.

[33] Peri PL, Ladd B, Pepper DA, Bonser SP, Laffan SW, et al (2011) Carbon (δ13C) and nitrogen (δ15N) stable isotope composition in plant and soil in Southern Patagonia's native forests. Global Change Biology 28: 1365–2486.

[34] Fang HJ, Yu GR, Cheng SL, Zhu TH, Wang YS, et al (2010) Effects of multiple environmental factors on CO(2) emission and CH(4) uptake from old-growth forest soils. Biogeosciences 7: 395–407.

[35] Lovett GM, Christenson LM, Groffman PM, Jones CG, Hart JE, et al (2002) Insect defoliation and nitrogen cycling in forests. BioScience 52: 335–341.

[36] Aber J, McDowell W, Nadelhoffer K, Magill A, Berntson G, et al (1998) Nitrogen saturation in temperate forest ecosystems. BioScience 48: 921–934.

[37] Gundersen P, Schmidt IK, Raulund-Rasmussen K (2006) Leaching of nitrate from temperate forests effects of air pollution and forest management. Environmental Reviews 14: 1–57.

[38] Aber JD, Goodale CL, Ollinger SV, Smith M-L, Magill AH, et al (2003) Is nitrogen deposition altering the nitrogen status of northeastern forests? BioScience 53: 375–389.

[39] Xu XL, Ouyang H, Richter A, Wanek W, Cao GM, et al (2011) Spatio-temporal variations determine plant-microbe competition for inorganic nitrogen in an alpine meadow. Journal of Ecology 99: 563–571.

[40] Zhang B, Liang C, He HB, Zhang XD (2013) Variations in Soil Microbial Communities and Residues Along an Altitude Gradient on the Northern Slope of Changbai Mountain, China. Plos One 8. 10.1371/journal.pone.0066184 PMC3679006 23776630

[41] Preston CM, Marshall VG, McCullough K, Mead DJ (1990) Fate of 15N-labelled fertilizer applied on snow at two forest sites in British Columbia. Canadian journal of forest research 20: 1583–1592.

[42] Garten C, Schwab A, Shirshac T (1998) Foliar retention of ^15^N tracers: implications for net canopy exchange in low-and high-elevation forest ecosystems. Forest ecology and management 103: 211–216.

[43] Garten Jr CT, Hanson PJ (1990) Foliar retention of ^15^ N-nitrate and ^15^ N-ammonium by red maple (*Acer rubrum*) and white oak (*Quercus alba*) leaves from simulated rain. Environmental and experimental botany 30: 333–342.

[44] Stachurski A, Zimka J (2002) Atmospheric deposition and ionic interactions within a beech canopy in the Karkonosze Mountains. Environmental pollution 118: 75–87. 11996385 10.1016/s0269-7491(01)00238-x

